# Contrast-enhanced ultrasound combined with elastic imaging for predicting the efficacy of concurrent chemoradiotherapy in cervical cancer: a feasibility study

**DOI:** 10.3389/fonc.2024.1301900

**Published:** 2024-04-03

**Authors:** Yujuan Ma, Xuebo Zhao, Xianxia Chen

**Affiliations:** ^1^ Tianshui Maternity and Child Healthcare Hospital, Tianshui, China; ^2^ Graduate School of Qinghai University, Xining, China; ^3^ Department of Ultrasound Medicine, Qinghai Provincial People’s Hospital, Xining, China

**Keywords:** contrast-enhanced ultrasound, ultrasonic elastography, cervical cancer, simultaneous chemoradiotherapy, predict

## Abstract

**Objective:**

Contrast-enhanced ultrasound (CEUS) and elastography are of great value in the diagnosis of cervical cancer (CC). However, there is limited research on the role of contrast-enhanced ultrasound combined with elastography in predicting concurrent chemoradiotherapy and disease progression for cervical cancer. The purpose of this study was to evaluate the feasibility of contrast-enhanced ultrasound combined with elastography and tumor prognosis.

**Methods:**

MRI was performed on 98 patients with cervical cancer before and after treatment. Before, during, and 1 week after the treatment, contrast-enhanced ultrasound and elastography were conducted, and the alterations of ultrasound-related parameters at each time point of the treatment were compared. The correlation between contrast-enhanced ultrasound combined with elastic imaging and oncological outcome was assessed.

**Results:**

There was no notable difference in overall clinical data between the complete remission (CR) group and the partial remission (PR) group (P>0.05). Before treatment, there were no statistically significant differences in elasticity score, time to peak (TTP), and peak intensity (PI) between the CR group and the PR group. However, there were no statistical differences in elastic strain ratio (SR) and area under the curve (AUC) before and after treatment between the CR group and the PR group, and there were also no statistical differences in the elastic strain ratio (SR) and area under the curve (AUC) of contrast-enhanced ultrasound parameters between the CR group and the PR group before and during treatment. There was a statistically significant difference after treatment (P<0.05).At present, the follow-up of patients is about 1 year, 7 patients were excluded due to loss to follow-up, and 91 patients were included in the follow-up study. Through the review of the cases and combined with MRI (version RECIST1.1) and serology and other related examinations, if the patient has a new lesion or the lesion is larger than before, the tumor marker Squamous cell carcinoma antigen (SCC-Ag) is significantly increased twice in a row, and the patient is divided into progressive disease (PD). Those who did not see significant changes were divided into stable disease (SD) group. The relationship between clinical characteristics, ultrasound parameters and disease progression in 91 patients was compared. There was no significant difference in age and clinical stage between the two groups (P>0.05), but there was a significant difference in the elevation of tumor marker squamous cell carcinoma antigen (SCC-Ag) between the two groups (P<0.05).With the growth of tumors, TTP decreased, elasticity score and PI increased, and the difference was statistically significant (P<0.05). The AUC of SCC-Ag was 0.655, the sensitivity was 85.3%, and the specificity was 45.6%.The AUC, sensitivity and specificity of ultrasound parameters combined with SCC-Ag predicted disease progression was 0.959, 91.2% and 94.8%.

**Conclusions:**

Using contrast-enhanced ultrasound and elastography to predict the efficacy and disease progression of concurrent chemoradiotherapy is feasible. In addition, the combination of SCC-Ag with contrast-enhanced ultrasound and elastography can further enhance the efficiency of predicting disease progression.

## Introduction

Cervical cancer (CC) is the second most common gynecologic malignant epithelial tumor in women worldwide (1),In most patients, the symptoms are insidious and difficult to detect at the beginning of the disease, and the stage of cervical cancer is already in the middle and advanced stages at the time of presentation (2). Therefore, annual cervical cancer screening for young and middle-aged women can detect cervical lesions early and intervene in time, which is the key to reducing patient mortality and improving patient outcomes (3). The 2019 edition of the NCCN Clinical Practice Guidelines (4) for Cervical Cancer indicates that synchronous chemoradiotherapy (CCRT) is used for patients with stage ⅡB ~IVA cervical cancer. Squamous cell carcinoma antigen (SCC-Ag) is a protein that is elevated in most patients with cervical squamous cell carcinoma (5). Studies have shown that (6, 7) the change in SCC-Ag levels is not only related to tumor size but is also one of the most important diagnostic and prognostic markers for CC. Therefore, the assessment of SCC-Ag levels is essential for clinicians to treat them in a timely manner (8, 9).

At present, the clinical treatment of Tumors is mainly judged based on the principle of Response Evaluation Criteria In Solid tumors 1.1(RECIST 1.1) (10). However, MRI is relatively expensive. The examination time is long, especially for patients wearing metal foreign bodies in the body is not suitable. Compared with MRI, ultrasound technologies such as ultrasonic elastography (USE) (11) and Contra-enhanced ultrasound (CEUS) (12) are more and more widely used in clinical practice because of their short examination time, relatively low cost and real-time dynamic observation of lesions. As a supplement to routine ultrasound, USE has been the focus of much medical research. USE has been thoroughly studied in thyroid, breast, liver and lymph node pathology (13). Meanwhile, USE has also been studied in differential diagnosis of cervical cancer, evaluation of infiltration degree, clinical staging and the assessment of treatment outcomes (14). After CCRT is applied to patients with advanced cervical cancer, a large number of tumor cells will undergo apoptosis, cleavage and other changes, the compliance of the tumor region will be improved, the elastic strain rate ratio (SR) will gradually decrease, and eventually the hardness of cervical cancer lesions will decrease. Therefore, by dynamically monitoring the changes of lesion elasticity score and SR during cervical cancer treatment, USE can play a better predictive role in the evaluation of CCRT efficacy (15). CEUS is a well-established clinical technique for the assessment of liver lesions (16). By drawing a time-intensity curve (TIC), CEUS extracts and analyses CEUS-related parameters to provide key information for the diagnostic stage and efficacy assessment of tumors. However, its application value in the evaluation of gynecological diseases remains to be proven (17), and there are few studies on the application of CEUS in cervical cancer (18–20).

Compared with MRI, CEUS combined with USE technique is more suitable for long-term follow-up of patients. Therefore, this study will explore the feasibility of CEUS and USE technology in predicting the efficacy and disease progression of concurrent chemoradiotherapy for cervical cancer.

## Materials and methods

### Research object

The study protocol was approved by the Institutional Review Committee of the Qinghai Provincial People’s Hospital (2021–72) and the patient’s written informed consent was obtained. Between March 2021 and February 2022, we prospectively enrolled 98 patients with pathologically confirmed cervical cancer who were scheduled to receive CCRT in our hospital. Inclusion criteria: (1) age > 18 years old; (2) Cervical cancer confirmed by pathology; (3) The clinical stages were in stage IIB - IVA; (4) The heart, liver, lung and other functions are normal. Exclusion criteria: (1) liver and kidney failure; (2) Abnormal blood system; (3) combined with other sites of malignant tumors; (4) Allergic to chemotherapy drugs; (5) had metastasized at the time of enrollment; (6) Pregnancy or lactation.

### CCRT

All patients were combined with radiotherapy and systemic chemotherapy for about 4 cycles, which could be adjusted according to the specific conditions of the patients. If the patient has nausea, vomiting and other reactions during treatment, timely intervention should be conducted and the patient’s blood routine and other indicators should be tested. Radiotherapy options include external beam radiotherapy (EBRT) and intracavitary high dose rate (HDR) brachytherapy. External beam radiotherapy: 5 times/week, 1 time/day, 2.0 ~ 2.2 Gy/time; The total dose does not exceed 55Gy; Meanwhile, the chemotherapy regimen was as follows: intravenous infusion of cisplatin (30mg/m2) and paclitaxel (60mg/m2) once a week. HDR brachytherapy was started in the last week of EBRT, and internal irradiation A point was set after the end of external irradiation, 5 ~ 6 Gy/time, once/day, 6 times/week, and the total dose did not exceed 35Gy. The definition of point A follows the recommendations of the American Society for Brachytherapy (21).

### Efficacy evaluation and follow-up

The 3.0T MRI scanner of Siemens in Germany was used to perform MRI examination on the patients included in the study one week before and after concurrent chemoradiotherapy. The maximum diameter of cervical cancer lesions for three consecutive times was measured and the average value was recorded. Two physicians who have been engaged in gynecological MRI diagnosis for more than 10 years were used to evaluate the efficacy of solid tumor by the evaluation criteria 1.1 (RECIST 1.1). The efficacy was divided into four groups: (1) Complete remission (CR) group: the tumor completely disappeared; ②Partial remission (PR) group, the maximum tumor diameter was reduced, and the reduction rate was ≥30%; ③ Disease progression (PD) group; The maximum diameter of the tumor increased, the rate of change increased by ≥20% or new lesions appeared. In the stable disease (SD) group, the maximum diameter change rate of the tumor was between PR and PD.

Relevant data files were established for patients at the time of the first examination, and follow-up was conducted for about 1 year starting from the initial examination of patients, and follow-up was generally conducted at an interval of 3 to 6 months. Clinical case data were consulted. Through ultrasound examination, if new lesions were found in patients or the maximum diameter of lesions was larger than before, and the value of SCC-Ag was significantly higher than before for two consecutive reviews, indicating disease progression, patients were included in PD group, whereas patients with no significant changes in lesions and SCC-Ag were included in SD group. All patients underwent contrast-enhanced ultrasound (CEUS) and elastography before treatment, and the ultrasonic-related parameters were recorded.

### USE examination and image analysis

**Figure 1:**
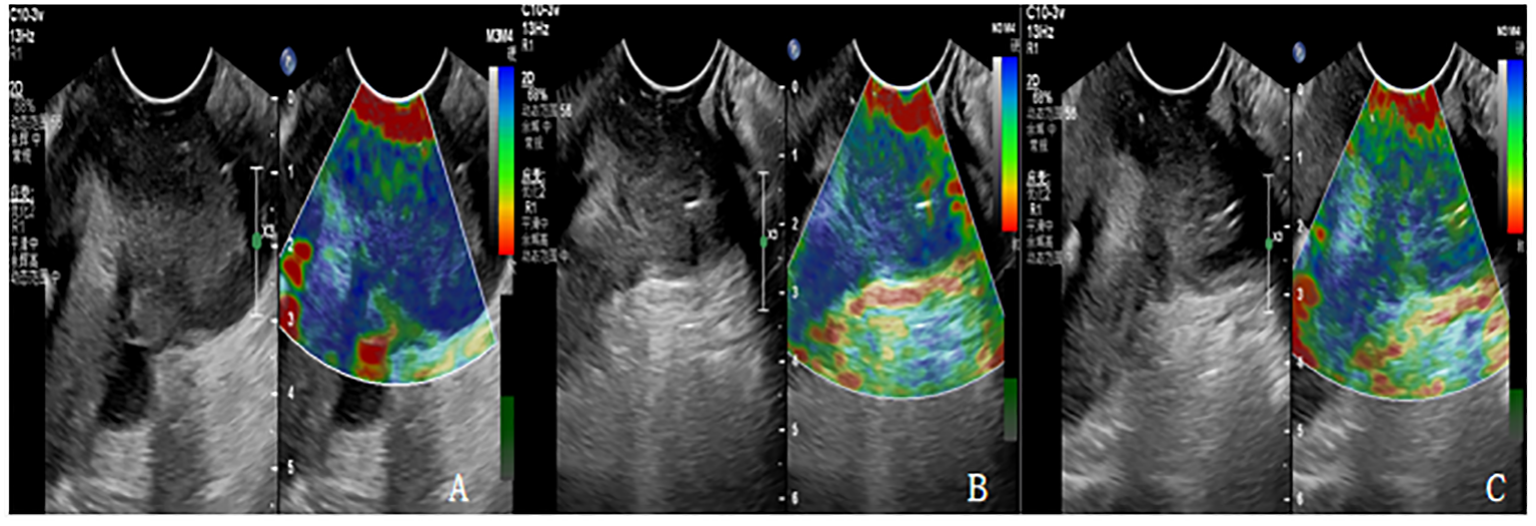
**(A-C)** are the Ultrasonic elastography of patients with cervical cancer at each time point of CCRT. **(A)** The lesion had hard elasticity and was basically blue, with an elasticity score of 5 and an SR value of 4.3. **(B)** The lesion had soft elasticity, blue in the center and a little green around it. The elasticity score was 4 points and SR value was 3.1. **(C)** The lesion had soft elasticity and appeared blue and green, with an elasticity score of 3 and an SR value of 2.4.

EPIQ Elite W color Doppler ultrasound diagnostic instrument (equipped with elastic imaging analysis software) was used to select an intracavity probe model C10-3V with a frequency of 5MHz for examination. USE examination (**Figure 1**) was performed about one week before, during and after CCRT. First of all, the bladder was emptied before examination and the bladder lithotomy position was maintained. Then, the intracavity probe with condom protection outside was placed at the curvature of the vagina for routine ultrasound examination. The best position with clear display of cervical cancer lesions was selected, and then the USE examination was selected. The selection range of cervical cancer lesions in the image frame should be expanded as far as possible. When the mass is too large to be selected, segmented sampling should be carried out to ensure that the best sampling location of USE is selected, the elastic images are clear and the dynamic images of USE are saved. When the images were frozen for observation, a 5 mm diameter ROI was selected for tumor lesions and normal tissue around the same layer, and the elasticity score was visually measured and recorded. The measuring point was measured three times and the average value was taken. Meanwhile, the relationship between the internal opening of the cervix and the vaginal fornix was recorded. Note that the maximum cross-section of the lesion was taken for analysis in each assessment. The ultrasound examination of all patients was performed by two persons with more than 10 years of professional experience, and the relevant medical records and other imaging results were not presented during the examination.

According to the elastic imaging images, the lesions were divided into 5 and (from soft to hard): a score of 1 indicated that most lesions were green; A score of 2 indicates that the green area of the lesion is more than 90%, and a small amount of blue is seen in the center. A score of 3 indicates that the lesion area is mixed with blue and green, and the proportion is similar. A score of 4 indicates that the lesion area is mostly blue, with only a little green around it. A score of 5 indicates that the lesion is mostly blue. If the score is 1-3, the lesion is considered benign. If the score is 4-5, the lesion is considered malignant.

Strain ratio (SR) is the strain ratio of the lesion to normal tissue at the same level, and the method of operation is as follows: When the tumor size and shape are clear, the sample frame is enlarged to cover the entire tumor area. Using 5mm as the diameter frame of the lesion area and the same level of normal cervical tissue around the lesion, the elasticity analysis software can automatically calculate the SR value of the lesion and the normal tissue on the same side.

### CEUS examination and image analysis

**Figure 2:**
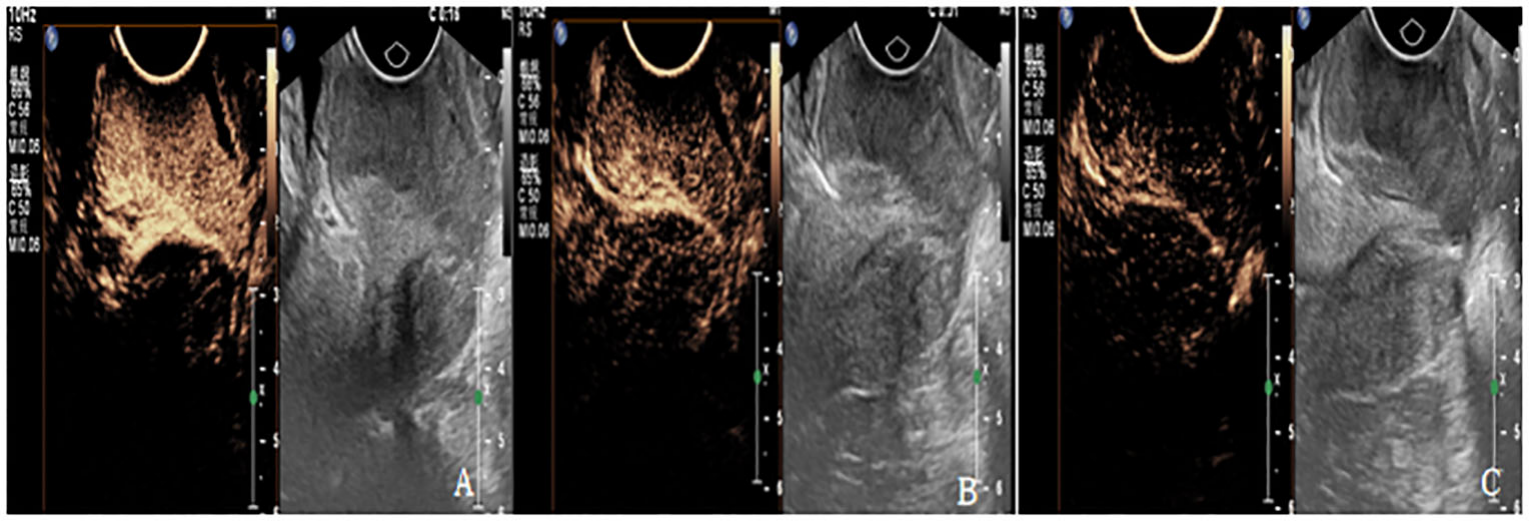
**(A-C)** are CEUS images of patients with cervical cancer at each treatment time point of CCRT. **(A)** The lesion showed high enhancement with PI of 17.2dB, TTP of 11S and AUC of 1120dB.s. **(B)** The lesions showed moderate enhancement, with PI of 11dB, TTP of 23S, and AUC of 984dB.S. **(C)** The lesion showed weak enhancement with PI of 5.3 dB, TTP of 53S, and AUC of 453dB.s.

Philips EPIQ Elite W color Doppler ultrasound diagnostic instrument (TIC curve analysis application software) was used for examination with intracavitary probe model C10-3V at a frequency of 5MHz, and CEUS (**Figure 2**) was performed about one week before, during and after concurrent chemoradiotherapy. Switching to contrast mode at the end of each elastography session, SonoVue, produced by Bracco, Italy, was used as the ultrasound contrast agent. Saline (5ml) was injected into a bottle containing SonoVue and mixed. SonoVue suspension (2.0ml) was then administered via an elbow vein, followed by 5ml of normal saline. The optimal section of blood perfusion was selected, and synchronized timing was started after the mode of low-mechanical index CEUS was switched to. Real-time 2D and CEUS images appeared on the screen, and the situation of contrast agent entering and exiting at the lesion site was dynamically observed. The CEUS video was saved after 180S. Analysis was conducted according to the perfusion characteristics of contrast agent in the lesion area and the mode of entering and exiting the lesion. At the same time, the lesion and the normal tissue frame at the same level were analyzed with 5mm as the diameter, and TIC curves were automatically generated with one click. The contrast parameter values in the upper left corner were recorded, including peak time, peak intensity and area under the curve. The ultrasound examination of all patients was conducted by two persons with more than 10 years of work experience, and the relevant medical records and other imaging results were not presented during the examination.

### Statistical analysis

In this study, SPSS 25.0 software was used to analyze the collected data, and the Shapiro-wilk test was used to test the normality of the data, the normally distributed continuous data was expressed as (x ± s), the t-test was used for comparison between groups, the non-normally distributed continuous data was expressed as M (IQR), and the Mann-Whitney U test was used for comparison between groups. The X^2^ test was used for the counts, and the data were expressed in n (%). Univariate and multivariate logistic regression analyses were performed on the clinical characteristics and ultrasound multiparameters of patients included in the follow-up studies and whether the patients had disease progression. The diagnostic performance of ultrasound parameters and clinical features combined with ultrasound multiparametric was compared, and the ROC curve was plotted. P<0.05 represents a statistically significant difference.

## Results

### General clinical data of the patient

Currently, 98 patients with advanced cervical cancer who were to receive CCRT were included, all of whom were squamous cell carcinoma. The patients were divided into CR group (23 cases) and PR group (75 cases), ranging in age from 29 to 71 years old, and no significant statistical difference was found in general clinical data between the two groups (P > 0.05) (**Table 1**).

**Table 1:** Comparison of clinical data of cervical cancer patients in CR group and PR group (x ± s).

	CR group (n=23)	PR group (n=75)	*X^2^/t*	*P*
Age (years)	51.70 ± 9.72	53.39 ± 10.18	-0.704	0.483
BMI (kg/m^2^)	22.73 ± 3.24	22.12 ± 2.28	0.869	0.421
Duration of treatment (week)	5.54 ± 1.11	5.83 ± 1.03	-1.174	0.243
Stage			1.928	0.165
Ⅰ	12 (52.20)	34 (45.31)		
Ⅱ	11 (47.82)	29 (38.74)		
Ⅲ	0 (0.0)	12 (20.0)		

### Comparison of elasticity scores between CR group and PR group at each treatment time point

The comparison of the changes of elastic scores of cervical cancer patients in CR group and PR group at various time points of treatment (**Table 2**) showed that there was no significant statistical difference in elastic scores before treatment (P > 0.05), and there were significant statistical differences during and after treatment. With the improvement of treatment, the overall lesion elasticity score showed a downward trend.

**Table 2:** Comparison of elastic scores of cervical cancer patients in CR group and PR group at each treatment time point [M (IQR)].

Group	Treatment time point		
	Before treatment (points)	Under treatment (points)	After treatment (points)
CR group (n=23)	5 (1)	3 (1)	2 (1)
PR group (n=75)	5 (1)	4 (1)	3 (0)
*Z*	1.930	12.232	52.717
*P*	0.165	0.000	0.000

### Comparison of SR at each treatment time point between CR group and PR group

The comparison of SR changes of cervical cancer patients in CR group and PR group at treatment time points (**Table 3**) showed no significant statistical difference between the two groups before and during treatment (P >0.05). There were significant differences after treatment (P< 0.05). With the improvement of treatment, SR decreased significantly, but there was no significant difference before and during treatment.

**Table 3:** Comparison of SR of cervical cancer patients in CR group and PR group at each treatment time point (x ± s).

Group	Treatment time point		
	Before treatment (points)	Under treatment (points)	After treatment (points)
CR group (n=23)	4.43 ± 0.30	3.45 ± 0.28	1.84 ± 0.61
PR group (n=75)	4.48 ± 0.22	3.50 ± 0.21	2.49 ± 0.27
*t*	-0.747	-0.833	-4.969
*P*	0.457	0.411	0.000

### Comparison of TTP at each treatment time point between CR group and PR group

The comparison of TTP changes of cervical cancer patients in CR group and PR group at each time point of treatment (**Table 4**) showed no significant statistical difference between the two groups before treatment (P > 0.05). There were significant differences during and after treatment (P<0.05). With the improvement of treatment, TTP showed an overall upward trend. Compared with before treatment, TTP in CR group increased faster than that in PR group after treatment.

**Table 4:** Comparison of TPP at various time points between cervical cancer patients in CR group and PR group (x ± s).

Group	Treatment time point		
	Before treatment (S)	Under treatment (S)	After treatment (S)
CR group (n=23)	17.53 ± 3.65	29.08 ± 4.52	73.12 ± 23.29
PR group (n=75)	18.23 ± 4.35	26.41 ± 5.19	55.85 ± 11.40
*t*	-0.701	-2.761	3.433
*P*	0.485	0.007	0.002

### Comparison of PI at each treatment time point between CR group and PR group

The comparison of PI changes of cervical cancer patients in CR group and PR group at each time point of treatment (**Table 5**) showed no significant statistical difference before treatment (P > 0.05). There were significant differences between the two groups during and after treatment (P< 0.05). With the improvement of treatment, the lesion PI in CR group decreased significantly than that in PR group.

**Table 5:** Comparison of PI values at different time points between CR group and PR group for cervical cancer patients (x ± s).

Group	Treatment time point		
	Before treatment (dB)	Under treatment (dB)	After treatment (dB)
CR group (n=23)	17.48 ± 0.90	14.35 ± 1.39	7.74 ± 2.93
PR group (n=75)	17.11 ± 0.97	15.05 ± 1.43	9.67 ± 3.32
*t*	1.619	-2.063	-2.552
*P*	0.109	0.042	0.013

### Comparison of AUC at each treatment time point between CR group and PR group

The comparison of AUC changes of cervical cancer patients in CR group and PR group at various time points of treatment (**Table 6**) showed no significant statistical difference between the two groups before and during treatment (P > 0.05). There was a significant difference after treatment (P < 0.05). With the improvement of treatment, PI in CR group decreased significantly than that in PR group.

**Table 6:** Comparison of AUC values of cervical cancer patients in CR group and PR group at different time points (x ± s).

Group	Treatment time point		
	Before treatment (dB.S)	Under treatment (dB.S)	After treatment (dB.S)
CR group (n=23)	1110.26 ± 129.79	501.06 ± 138.76	294.27 ± 61.97
PR group (n=75)	1152.18 ± 171.82	516.68 ± 96.35	359.71 ± 74.72
*t*	-1.078	-0.612	-3.814
*P*	0.284	0.542	0.000

### The relationship between clinical features and disease progression was followed up

The clinical characteristics and disease progression of 91 patients included in follow-up were compared (**Table 7**). SCC-Ag was divided into yes (significantly increased after two consecutive reviews) and no (no significant increase after two consecutive reviews). There was no statistically significant difference in age and clinical stage (P≥0.05), while there was a statistically significant difference in the increase of SCC-Ag (P < 0.05). There were more patients with elevated SCC-Ag in PD group than in SD group.

**Table 7:** Comparison of clinical features and disease progression in follow-up patients (x ± s).

	SD group (n=57)	PD group (n=34)	*t/X^2^*	*P*
Age (years)	51.97 ± 10.77	55.44 ± 7.85	-1.639	0.105
Stages			1.928	0.165
Ⅰ	16 (28.07)	7 (20.59)		
Ⅱ	26 (45.61)	8 (23.53)		
Ⅲ	11 (19.30)	14 (41.18)		
Ⅳ	4 (7.02)	5 (14.71)		
SCC-Ag			8.191	0.004
Yes	32 (56.14)	29 (85.29)		
No	25 (43.86)	5 (14.17)		

### Relationship between ultrasound parameters and disease progression in follow-up patients

**Figure 3:**
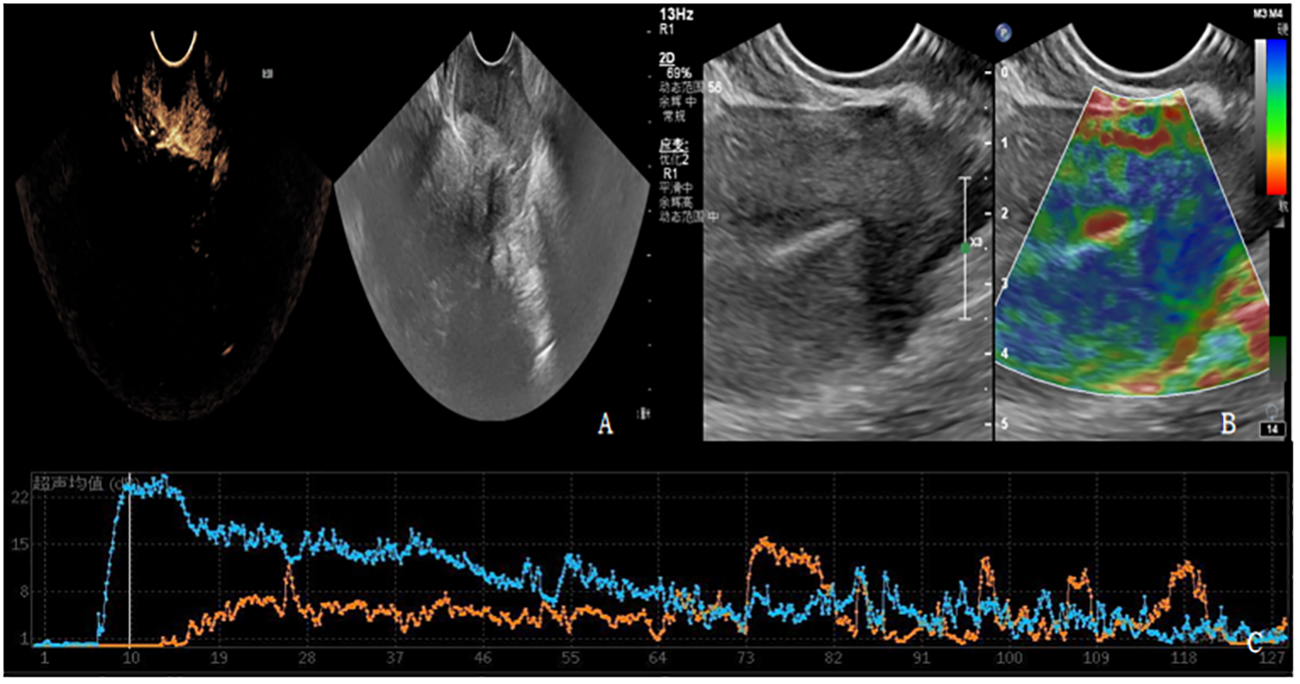
**(A-C)** shows contrast-enhanced ultrasound and ultrasound elastography of PD patients. **(A)** Lesions showed moderate to high degree of enhancement. **(B)** The lesion had stiff elasticity, the center was blue and green, and blue was the majority. The elasticity score was 4 points, and the SR value was about 3.8. **(C)** TIC curve drawn after focal angiography. Blue represents the TIC curve of the lesion, and orange represents the TIC curve of normal tissue at the same level of the lesion. The lesion PI was 22dB, TTP was 10S, and AUC was 1124dB.S.

**Figure 4:**
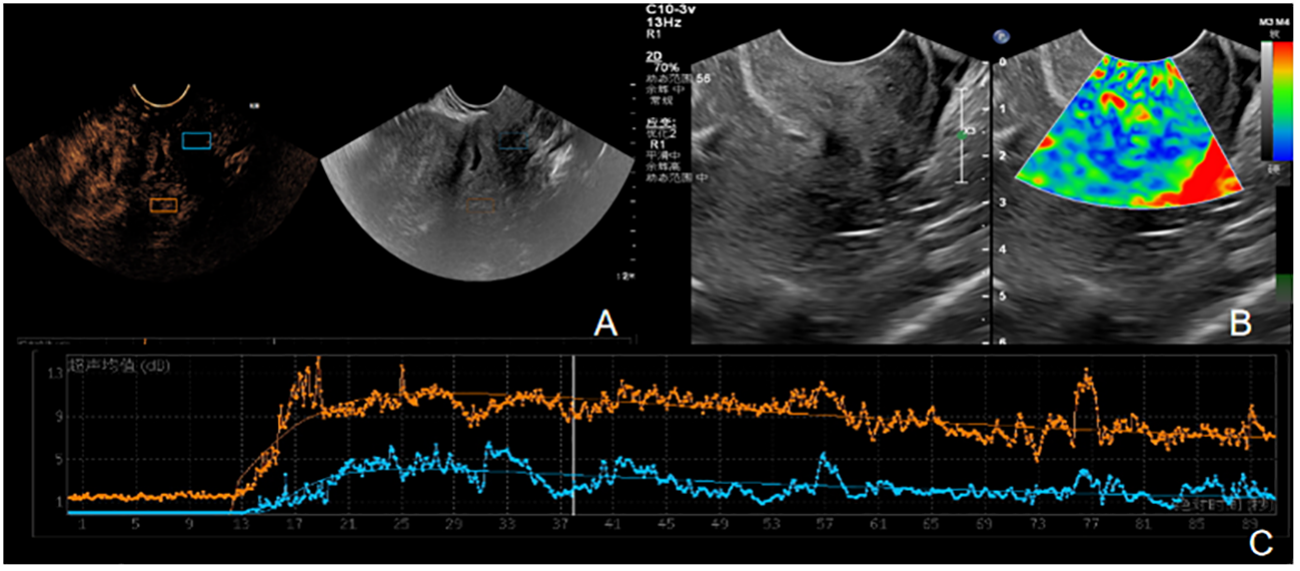
**(A-C)** is the contrast ultrasound and elastography of SD patients. **(A)** There was no obvious enhancement in the lesion area. **(B)** The elasticity of the lesion was soft, the center was blue and green, and blue was the majority. The elasticity score was 2, and the SR value was about 1.6. **(C)** TIC curve drawn after angiographic imaging of the lesion area. Blue represents TIC curve of the lesion area, and orange represents TIC curve of normal tissue at the same level of the lesion. The lesion PI was 9dB, TTP was 28S, and AUC was 521 DB.s.

Patients in PD group and SD group underwent contrast-enhanced ultrasound and elastography (**Figures 3**, **4**). The ultrasound parameters with sensitive changes before and after treatment were compared with the prognosis of disease progression (**Table 8**), and it was found that with the disease progression of patients, elasticity score and PI value increased, while TTP decreased, with statistically significant differences (P < 0.05).

**Table 8:** Comparison of ultrasound parameters and disease progression in follow-up patients (x ± s)/[M (IQR)].

Group	SD group	PD group	*t/Z*	*P*
Elasticity score (score)	3 (2)	4 (1)	-5.043	0.000
TTP (S)	28.49 ± 10.98	17.01 ± 5.35	6.677	0.000
PI (dB)	8.49 ± 2.55	12.43 ± 2.60	-7.063	0.000

### Logistic regression analysis of SCC-Ag and ultrasound parameters

Logistic regression analysis was performed on SCC-Ag, elasticity score, PI and TTP (**Table 9**). SCC-Ag, elasticity score, PI and TTP (all P<0.05), meaning that it has a significant relationship with whether the disease progresses. At the same time, SCC-Ag, elasticity score and PI (all OR values > 1) indicated that the increase of SCC-Ag, elasticity score and PI values, the greater the possibility of disease progression. The OR value of TTP < 1 indicates that the TTP value of patients is negatively correlated with the disease progression, and the lower the TTP value, the greater the possibility of disease progression. logistic regression equation was established:

**Table 9:** Logistic regression analysis of SCC-Ag and ultrasonic parameters.

index	*β*	S.E	Wald	*P*	*OR*	*95%CI*
Elasticity score (score)	1.214	0.285	18.173	0.000	3.366	1.926~5.880
PI (dB)	0.540	0.111	23.640	0.000	1.717	1.381~2.135
TTP (s)	-0.237	0.051	21.632	0.000	0.789	0.717~0.872
SCC-Ag (Yes VS No)	1.511	0.553	7.468	0.006	4.531	1.533~13.393

logistic (P) =-6.153 + 0.806× elasticity score +0.543×PI+ (-0.203) ×TTP+1.866×SCC-Ag. The likelihood ratio test of the regression model shows that P=0.00 and the observed goodness of fit value of Hosmer-Lemeshow is 6.605 (P=0.580).

### ROC curve of SCC-Ag and ultrasonic parameters predicting disease progression

**Figure 5:**
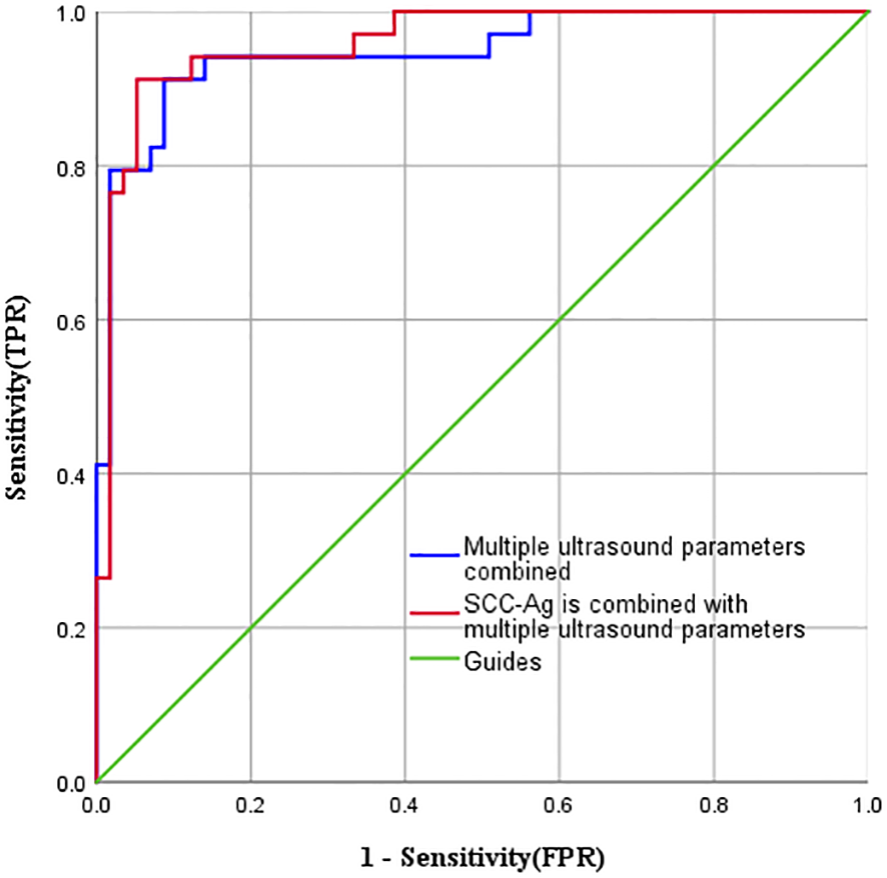
Multiple Ultrasonic parameters (elasticity score, PI, TTP), SCC-Ag and Multiple ultrasonic parameters (elasticity score, PI, TTP) combined to predict the ROC curve of disease progression.

The combined efficacy of SCC-Ag, ultrasonic parameters (elasticity score, PI, TTP), SCC-Ag and ultrasonic parameters (elasticity score, PI, TTP) in predicting disease progression was compared. The AUC of SCC-Ag in predicting disease progression was 0.655, the sensitivity was 85.3%, and the specificity was 45.6%. The AUC of ultrasonic parameters was 0.948, the sensitivity was 91.2%, and the specificity was 91.2%. The AUC of the combination of ultrasound parameters and SCC-Ag in predicting disease progression increased to 0.959, the sensitivity was 91.2% (same as that of the combination of ultrasound parameters), and the specificity increased to 94.8%. The ROC curve of multiple ultrasonic parameters (elasticity score, PI, TTP), SCC-Ag and multiple ultrasonic parameters (elasticity score, PI, TTP) combined to predict disease progression was plotted (**Figure 5**).

## Discussion

Cervical cancer (CC) is the second most common gynecologic malignant epithelial tumor in women worldwide. At present, CCRT is mostly used for patients with advanced cervical cancer. Studies have shown that (22) CCRT can significantly improve overall survival and progression-free survival in patients with advanced cervical cancer, but there are also varying degrees of toxicity and complications (23). Therefore, it is important to review the prognosis of patients through timely and effective follow-up, identify the factors that can predict the risk of recurrence, and prevent cervical cancer recurrence through early clinical follow-up intervention and further optimization of treatment (24).

USE is different from conventional ultrasound, which shows the acoustic properties of the lesion, while USE shows the biomechanical properties of the tissue, which are not related to each other (25). When the tissue is elastography-imaged, there are corresponding changes in the interior of the tissue, and the areas with high strain are blue, the areas with low strain are red, and the areas with moderate strain are green. For patients with advanced cervical cancer, the cervical tumor area is hard, the elasticity score is high, and the SR increases. As the tumor volume decreases after CCRT, the apoptosis and cleavage of tumor cells occur, and the elasticity of cervical cancer lesions decreases, and the elasticity score and SR also change. Therefore, by dynamically monitoring the changes of SR and elastic score before and after concurrent radiotherapy and chemotherapy of cervical cancer, USE can play a better predictive role in the evaluation of CCRT efficacy. Studies have shown that (26) after CCRT, the tumor volume of patients with advanced cervical cancer decreases and the compliance of tumor tissues increases, which is manifested as the decrease of tumor tissue hardness, elasticity score and SR. It has been reported that (15) early measurement in CCRT by elastography can detect changes in tumor hardness and predict the long-term prognosis of patients with locally advanced cervical cancer. At the same time, the study proved that SR has good diagnostic efficacy in distinguishing benign and malignant lesions. Compared with elastic score, SR has a higher accuracy in predicting cervical cancer, with a sensitivity of 90.9% and a specificity of 90.0% (27). Studies have shown that (28) through the USE examination before and after CCRT in cervical cancer patients, there is no significant difference in SR between the complete remission group and the partial remission group before treatment. As the disease improves, lesion hardness decreases and SR decreases, indicating that changes in SR before and after treatment can monitor and predict the treatment response of cervical cancer patients with CCRT. This can help clinicians optimize individual treatment and avoid unnecessary costs. Therefore, it can be seen from the above studies that USE can not only differentiate the diagnosis of benign and malignant lesions, but also predict the prognosis of cervical cancer CCRT. However, the USE technique also has limitations and cannot identify residual lesions in the cervix. CEUS has good agreement with pathology in the diagnosis and staging of cervical cancer, similar to MRI, and has certain advantages in showing small masses (29). CEUS quantitative analysis software draws TIC and extracts CEUS-related parameters to analyze CEUS parameters, providing key information for diagnostic staging and tumor response assessment (30). To date, there have been few studies on CEUS in cervical cancer (31), especially in evaluating the efficacy in cervical cancer. In this study, the combination of CEUS and USE technology was used to predict the efficacy of CCRT in patients with advanced cervical cancer. The combined examination can improve the assessment of prognosis and efficacy in patients with advanced cervical cancer and provide important reference information for clinical follow-up treatment.

In this study, CEUS was used to evaluate CCET patients before, during and after treatment. As CCRT treatment progressed in both the CR and PR groups, lesion TTP showed an overall upward trend. Compared to pre-treatment, TTP increased faster in the CR group than in the PR group. Similarly, the pre-treatment PI values of the CR and PR groups showed a decreasing trend with the progression of CCRT treatment. With the progression of CCRT treatment, the AUC in the CR and PR groups showed a decreasing trend, and the AUC in the CR group was more pronounced than that in the PR group. This study showed that PI and AUC after CEUS treatment were significantly lower than before treatment, while TTP was higher than before treatment. At the same time, this study showed that the elasticity scores and contrast-enhanced ultrasound parameters PI and TTP in CR and PR groups during and after treatment were statistically different (P<0.05), indicating that the elasticity score of USE and CEUS parameters PI and TTP are sensitive to treatment during the whole treatment process and have a clear effect on the prognosis of patients, which can be used as one of the effective indicators for prognosis evaluation.

According to clinical and related reports (32, 33), cervical cancer is closely related to SCC-Ag. If the level of SCC-Ag in a patient is significantly increased for two consecutive reviews, it indicates that the patient may have disease progression. After CCRT treatment, the lesions of patients with advanced cervical cancer will decrease or disappear, and the clinical symptoms and signs will also be reduced, but some patients have certain toxic complications, and new lesions may appear or the lesions will be larger than before if they are reexamined after a period of follow-up. At the end of this study, patients with advanced cervical cancer enrolled in CCRT were followed up for one year. By consulting clinical case data, including SCC-Ag and imaging examinations, the patients were classified into PD group and SD group respectively. First of all, this study compared patients’ SCC-Ag and ultrasound parameters sensitive to treatment before and after treatment with whether patients’ disease progressed. It was found that patients with increased SCC-Ag had a higher proportion of PD group, while patients in PD group had higher elasticity score and PI value, while lower TTP. Secondly, SCC-Ag, elasticity score, PI and TTP were included in logistic regression analysis. SCC-Ag, elasticity score and PI (mean OR value > 1) indicated that the higher the SCC-Ag, elasticity score and PI values of patients, the greater the probability of disease progression. The OR value of TTP < 1 indicates that the TTP value of patients is negatively correlated with the disease progression. The lower the TTP value, the greater the risk of disease progression. At the same time, logistic regression analysis showed that SCC-Ag, elastic score in ultrasound parameters, increase of PI, and decrease of TTP were risk factors for disease progression.

Finally, this study compared the diagnostic efficacy of SCC-Ag, ultrasound parameters (elasticity score, PI, TTP) and ultrasound parameters combined with SCC-Ag to predict disease progression in patients. Increased SCC-Ag predicted disease progression with an AUC of 0.655, sensitivity of 85.3% and specificity of 45.6%. The AUC of the ultrasound parameters predicting disease progression was 0.948, with sensitivity and specificity of 91.2%. When the ultrasound parameters were combined with SCC-Ag, the AUC increased to 0.959, the sensitivity was 91.2% and the specificity was 94.7%. Therefore, CEUS and ultrasound elastography have good application value in predicting disease progression in patients. When SCC-Ag is combined with ultrasound parameters, its specificity is significantly improved.

## Conclusions

Therefore, it is feasible and valuable to predict the curative effect of concurrent chemoradiotherapy and disease progression in cervical cancer using CEUS and elastography. Furthermore, SCC-Ag combined with ultrasound parameters can significantly improve the efficiency of predicting disease progression. Clinically, SCC-Ag and ultrasound parameters can be combined to predict the risk of disease progression in patients, so that patients with disease progression without obvious signs and symptoms can not only be detected and treated in time, but also their long-term survival can be prolonged.

## Data availability statement

The raw data supporting the conclusions of this article will be made available by the authors, without undue reservation.

## Ethics statement

The studies involving human participants are reviewed and approved by the Institutional Review Committee of Qinghai Provincial People’s Hospital and conducted in accordance with the Declaration of Helsinki. The studies were conducted in accordance with the local legislation and institutional requirements. The participants provided their written informed consent to participate in this study.

## Author contributions

XZ: Writing – review & editing, Visualization. YM: Writing – review & editing, Writing – original draft. XC: Writing – original draft, Writing – review & editing.
